# Genetic Analysis for Two Italian Siblings with Usher Syndrome and Schizophrenia

**DOI:** 10.1155/2012/380863

**Published:** 2012-10-04

**Authors:** Daniela Domanico, Serena Fragiotta, Paolo Trabucco, Marcella Nebbioso, Enzo Maria Vingolo

**Affiliations:** ^1^Department of Ophthalmology, “S. M. Goretti” Hospital, Sapienza University of Rome, Via G. Reni, 04100 Latina, Italy; ^2^Department of Ophthalmology, “A. Fiorini” Hospital, Sapienza University of Rome, Polo Pontino, Via Firenze, 04019 Terracina, Italy; ^3^Department of Sense Organs, Centre of Ocular Electrophysiology, Sapienza University of Rome, Viale del Policlinico 155, 00161 Rome, Italy

## Abstract

Usher syndrome is a group of autosomal recessive genetic disorders characterized by deafness, retinitis pigmentosa, and sometimes vestibular areflexia. The relationship between Usher syndrome and mental disorders, most commonly a “schizophrenia-like” psychosis, is sometimes described in the literature. The etiology of psychiatric expression of Usher syndrome is still unclear. We reported a case of two natural siblings with congenital hypoacusis, retinitis pigmentosa, and psychiatric symptoms. Clinical features and genetic analysis were also reported. We analyzed possible causes to explain the high prevalence of psychiatric manifestations in Usher syndrome: genetic factors, brain damage, and “stress-related” hypothesis.

## 1. Introduction

Usher syndrome represents a group of clinically variable and genetically heterogeneous disorders characterized by congenital sensorineural hearing loss, retinitis pigmentosa (RP), and sometimes vestibular areflexia [1]. Three clinical subtypes of Usher syndrome were recognized. Type I (USH1) is characterized by profound congenital deafness, prepubertal-onset retinitis pigmentosa, and vestibular dysfunction. Usher syndrome type II (USH2) is characterized by congenital mild to severe hearing loss, adolescent-onset retinitis pigmentosa, and no vestibular dysfunction. Usher syndrome type III (USH3) is characterized by rapidly progressive hearing loss. Age of onset of retinitis pigmentosa and degree of vestibular dysfunction are variable [2, 3]. To date, seven loci (USH1B-USH1H) and five genes for USH1 have been reported: USH1C, MYO7A, CDH23, PCDH15, and USH1G. Three genetic loci (USH2A, USH2C, and USH2D) and three genes (USH2A, GPR98, and DFNB31) have been identified in USH2. Mutations in USH2A gene on chromosome 1q41 are the most common mutations (85% of all cases with USH2). USH3 is caused by mutations in USH3A (clarin-1) gene, mapped on 3q21-q25 [4, 5]. Previous studies reported association between Usher syndrome and mental disorders, most commonly schizophrenia. Although Hallgren reported a prevalence of about 23% of psychotic disorders in individuals with Usher syndrome, other authors reported a prevalence of schizophrenia of only 4.5% [6, 7]. In addition, Dammeyer reported that 23% of individuals with Usher syndrome were affected by mental and behavioral disorders (such as mental retardation, anorexia nervosa, and ADHD) [8].

**Case 1**

A 26-year-old Caucasian female, born through an eutocic uncomplicated delivery, was first admitted to our department at the age of 23 complaining of gradual vision loss and hemeralopia in the last year.

**Figure 1:**
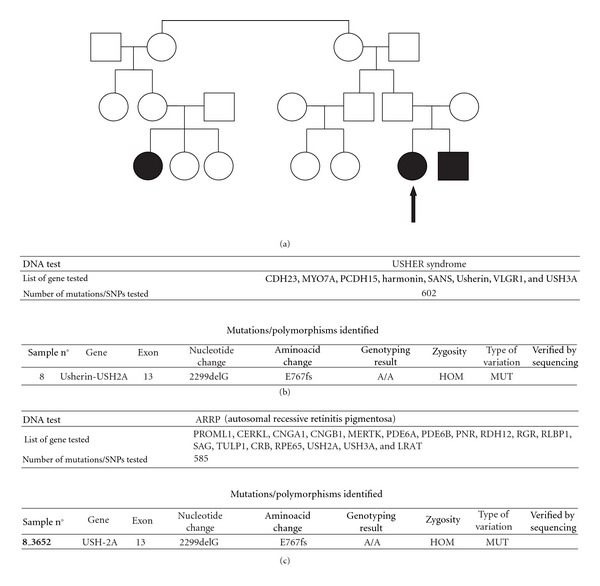


She had a history of bilateral hearing loss at 8 months of age, and her intelligence was normal; she was graduated from high school. Audiological examination showed bilateral sensorineural hearing loss. Her family history was positive: her youngest brother had similar symptoms, and her second cousin had hearing loss and retinopathy but no psychotic symptoms (Figure 1(a)). 

In her midteens, she showed behavioral changes characterized by irritability, reduced need for sleep, auditory hallucinations, and psychomotor agitation without negative symptoms. She was confused, inattentive, and presenting social isolation and impaired communication. She was admitted to the psychiatric unit for a mental evaluation. Neurological examination was normal. She also denied alcohol and illicit drug use. Treatment with risperidone 1 mg BID was initiated; it was well tolerated, and the symptoms were resolved. 

A completeophthalmologic examination was performed, including visual acuity, slit-lamp biomicroscopy, and dilated funduscopic examination. Best-corrected visual acuity (BCVA) was measured using a standard Snellen chart and then converted to a logarithm of the minimum angle of resolution (LogMAR). Written informed consent form was obtained for all diagnostic and therapeutic procedures employed. BCVA was 0.52 logMAR in the right eye (RE) and 0.39 logMAR in the left eye (LE). Results of slit-lamp examination were normal. Funduscopic examination revealed characteristic pigmentary changes of the retina, retinal arteriolar narrowing, and waxy pallor of the optic disc. Electroretinogram showed unrecordable rod responses, oscillatory potential, and markedly reduced mixed rod cone; and cone responses to a single-flash, and 30 Hz flicker were significantly delayed in implicit time and reduced in amplitude in both eyes. Visual field was measured with the Humphrey Field Analyzer (HFA), using program 30–2 (stimulus size III). The Humphrey visual field showed a complete peripheral visual field loss and less than 20-degree central visual fields. Molecular genetic testing was conducted from peripheral blood according to standard protocol, using a genotyping microarray to screen 602 mutations in 8 genes (CHD23, MYO7A, PCDH15, harmonin, SANS, Usherin, VLGR1, and USH3A). Furthermore, the autosomal recessive retinitis pigmentosa (ARRP) test was conducted, and the DNA was screened for 585 mutations in 18 genes. The analysis revealed homozygous mutation in USH2A gene and 2299delG in exon 13 (Figure 1(b)).

**Case 2**

Her 22-year-old brother, born at full term from eutocic delivery, was admitted to our department at the age of 19. He had a history of bilateral profound sensorineural hearing loss. Deafness was diagnosed at the age of five months, and vestibular function was normal. At the age of 7, he was diagnosed with Usher syndrome, and the diagnosis of retinitis pigmentosa was clinically made by an ophthalmologist.

His mental and physical development during infancy was normal; he had no trouble learning and graduated from secondary expert technical school. There was no known history of any other psychiatric disorders in his family. He also denied alcohol and illicit drug use. 

At the age of 11, he was diagnosed with attention deficit hyperactivity disorder (ADHD) by a psychiatrist and was treated for 4 years. At the age of 16, psychiatric disorder was suspected. His neurological examination was normal. Irritability, social isolation, psychomotor agitation, and visual and auditory hallucinations were noted. He walked back and forth in the house, had insomnia, isolated himself from his friends and family, and believed that others were plotting against him (delirium of persecution). He was diagnosed with paranoid schizophrenia and treated with Olanzapine with good response. At the age of 20, panic-like attacks and anxiety appeared, he did not become violent against his family and but showed obsessive behavior, depressive affects, and major anxiety. 

**Figure 2:**
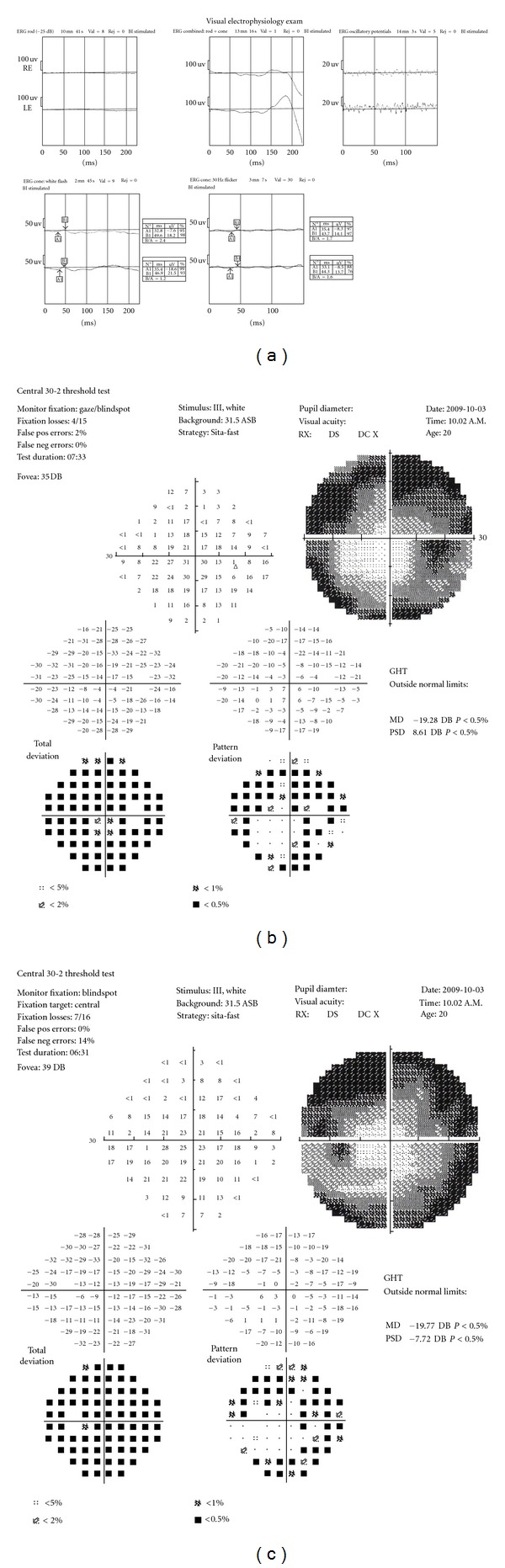


The patient was admitted to our department, and a complete eye examination was performed. BCVA was 0.52 logMAR in both eyes. Examination of the anterior segment, including lens and vitreous cavity, was unremarkable. Dilated fundus examination revealed a typical picture of retinitis pigmentosa more advanced than in his older sister. Electroretinogram showed unrecordable rod responses and mixed rod-cone and oscillatory potential, and cone responses to a single-flash and 30 Hz flicker were markedly reduced in amplitude, and the implicit time was significantly delayed in both eyes (Figure 2(a)). Humphrey Field Analyzer showed that visual field situated in the periphery of the 10° field was severely reduced (Figures 2(b)-2(c)). Genetic analysis was performed using the Usher syndrome and ARRP microarrays and revealed homozygous mutation of USH2A 2299delG (aminoacid change E767fs) in exon 13.

## 2. Discussion

To date, there were various reports in literature about associations between mental and behavioral disorders and RP syndromes, mostly in Usher syndrome. But there are conflicting data about the prevalence of psychotic illness in these patients. Hallgren [6] reported that psychosis was diagnosed in 26 of 114 persons (23% of cases) with Usher syndrome, whereas Nuutila [7] observed the prevalence of psychosis in 6 out of 131 patients (4.5% of cases). Dammeyer reported that 6 out of 26 patients (23%) were diagnosed with mental or behavioral disorders, including schizophrenia, mild and severe mental retardation, atypical autism, and conduct disorder. In this study, only 1 patient was diagnosed with schizophrenia, according to results reported by Nuutila, Grøndahl, and Mjøen [8]. The etiopathogenesis of the psychotic symptoms in Usher syndrome remains unclear. Three possible explanations for the high prevalence of psychotic illness in Usher syndrome should be considered: first, gene or genes that may predispose to both RP and psychosis. This mechanism was suspected because of cases that reported psychotic illness associated with Usher syndrome in the same family [9, 10]. To date, there is no evidence of the loci being on the same chromosome. However, it does not exclude the possibility of linkage, and further family studies are required [11]. 

 A second possible explanation is that Usher syndrome involves multiple brain regions. To confirm this, various abnormalities in central nervous system were found in patients with Usher syndrome. Computed tomographic (CT) and magnetic resonance imaging (MRI) studies showed the presence of cerebellar and cerebral atrophy, hypoplasia of corpus callosum and dilatation of fourth ventricle, decrease in intracranial volume with an increase in the size of the subarachnoid spaces, and arachnoid cyst together with cavum septum pellucidum and vergae. In view of these findings, the pathophysiology of Usher syndrome is complex, involving not only optic and auditory nerves, but also the CNS diffusely [11–14]. 

A third possible explanation is based on the theory that psychosis is stress-related response due to progressive sensory impairments. This theory is supported by the fact that visual or auditory impairment is associated with higher rate of depression, suicidal behavior, psychological stress, and social handicap. In addition as occurs in Charles Bonnet syndrome, characterized by visual loss and complex visual hallucinations, it may be related to abnormal central processing [9, 15]. Sufficiently prolonged isolation from society or deprivation of sensory stimuli can produce mental abnormalities in the form of hallucinations, anxiety states, depression, and paranoid symptoms, as discussed by Ziskind [16], who reported the higher prevalence of psychotic manifestations after extraction of the cataract. Indeed, in the past the occlusion of both eyes after cataract surgery was practiced to reduce the ocular movements that could interfere with the surgical wound healing. A small number of patients developed postoperative psychoses. The elimination of visual stimuli may produce a break with reality, and the restoration of vision usually was sufficient to remove psychotic symptoms. 

We reported the cases of two siblings with Usher syndrome associated with psychotic symptoms. To the best of our knowledge, this is the first study that reported the genetic analysis, demonstrating the association between usherin (USH2A) gene and psychotic manifestations. In our opinion, the pathogenesis of psychotic illness is complex and, probably, multifactorial. Multiple genes and environmental factors, such as isolation, sensory deprivation, anxiety, and stress-related disease, may be involved. Nevertheless, in our view, there is an overwhelming evidence of a strong genetic component. Indeed in our report and also in the literature [9, 10, 17], in most cases, the presence of psychotic symptoms occurs in more members of a family and often in patients with suspected diagnosis of Usher syndrome type II. However, only in our work was established the diagnosis with certainty by genetic analysis. For these reasons, further genetic studies are needed to explore the possibility of candidate genes in linkage regions. 

## References

[1] Hope CI, Bundey S, Proops D (1997). Usher syndrome in the city of Birmingham—prevalence and clinical classification. *British Journal of Ophthalmology*.

[2] Keats BJB, Savas S (2004). Genetic heterogeneity in Usher syndrome. *American Journal of Medical Genetics*.

[3] Tsilou ET, Rubin BI, Caruso RC (2002). Usher syndrome clinical types I and II: could ocular symptoms and signs differentiate between the two types?. *Acta Ophthalmologica Scandinavica*.

[4] Vozzi D, Aaspõllu A, Athanasakis E (2011). Molecular epidemiology of Usher syndrome in Italy. *Molecular Vision*.

[5] Bolz HJ, Roux A-F (2011). Clinical utility gene card for: Usher syndrome. *European Journal of Human Genetics*.

[6] Hallgren B (1959). Retinitis pigmentosa combined with congenital deafness; with vestibulo-cerebellar ataxia and mental abnormality in a proportion of cases: a clinical and genetico-statistical study. *Acta Psychiatrica Scandinavica, Supplementum*.

[7] Nuutila A (1970). Dystrophia retinae pigmentosa—dysacusis syndrome (DRD): a study of the Usher- or Hallgren syndrome. *Journal de Genetique Humaine*.

[8] Dammeyer J (2012). Children with Usher syndrome: mental and behavioral disorders. *Behavioral and Brain Functions*.

[9] McDonald C, Kenna P, Larkin T (1998). Retinitis pigmentosa and schizophrenia. *European Psychiatry*.

[10] Wu CY, Chiu CC (2006). Usher syndrome with psychotic symptoms: two cases in the same family. *Psychiatry and Clinical Neurosciences*.

[11] Koizumi J, Ofuku K, Sakuma K, Shiraishi H, Iio M, Nawano S (1988). CNS changes in Usher’s syndrome with mental disorder: CT, MRI and PET findings. *Journal of Neurology Neurosurgery and Psychiatry*.

[12] Bloom TD, Fishman GA, Mafee MF (1983). Usher’s syndrome. CNS defects determined by computed tomography. *Retina*.

[13] Schaefer GB, Bodensteiner JB, Thompson JN, Kimberling WJ, Craft JM (1998). Volumetric neuroimaging in Usher syndrome: evidence of global involvement. *American Journal of Medical Genetics*.

[14] Demir HD, Deniz FE, YardIm H (2010). A rare brain developmental anomaly in a patient with Usher’s syndrome. *International Ophthalmology*.

[15] Waldeck T, Wyszynski B, Medalia A (2001). The relationship between Usher’s syndrome and psychosis with capgras syndrome. *Psychiatry*.

[16] Ziskind E (1958). Isolation stress in medical and mental illness. *Journal of the American Medical Association*.

[17] Hess-Röver J, Crichton J, Byrne K, Holland AJ (1999). Diagnosis and treatment of a severe psychotic illness in a man with dual severe sensory impairments caused by the presence of Usher syndrome. *Journal of Intellectual Disability Research*.

